# Dynamic covalent synthesis†

**DOI:** 10.1039/d3sc05343a

**Published:** 2023-12-11

**Authors:** Fabien B. L. Cougnon, Artur R. Stefankiewicz, Sébastien Ulrich

**Affiliations:** a Department of Chemistry and Nanoscience Centre, University of Jyväskylä Jyväskylä Finland fabien.b.l.cougnon@jyu.fi; b Centre for Advanced Technology and Faculty of Chemistry, Adam Mickiewicz University Poznań Poland ars@amu.edu.pl; c Institut des Biomolécules Max Mousseron (IBMM), Université de Montpellier, CNRS, ENSCM Montpellier France sebastien.ulrich@cnrs.fr

## Abstract

Dynamic covalent synthesis aims to precisely control the assembly of simple building blocks linked by reversible covalent bonds to generate a single, structurally complex, product. In recent years, considerable progress in the programmability of dynamic covalent systems has enabled easy access to a broad range of assemblies, including macrocycles, shape-persistent cages, unconventional foldamers and mechanically-interlocked species (catenanes, knots, *etc.*). The reversibility of the covalent linkages can be either switched off to yield stable, isolable products or activated by specific physico-chemical stimuli, allowing the assemblies to adapt and respond to environmental changes in a controlled manner. This activatable dynamic property makes dynamic covalent assemblies particularly attractive for the design of complex matter, smart chemical systems, out-of-equilibrium systems, and molecular devices.

## Abstract 2

Toward organic self-synthesis using encoded synthons and programmed processes: we summarize here the design principles and the recent achievements which illustrate the power of dynamic covalent synthesis for making complex covalent architectures.

## Graphical abstract

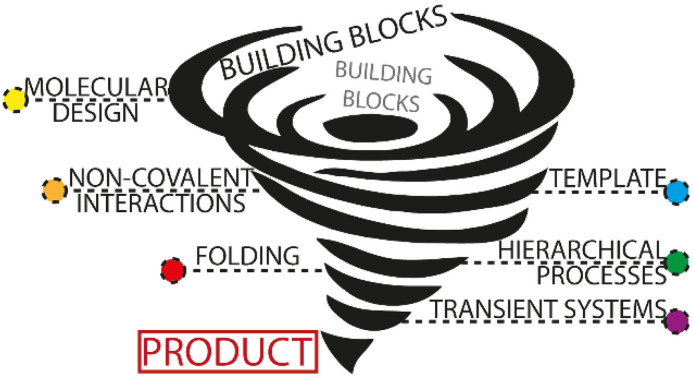

## 1. Introduction

Covalent and non-covalent self-assembly are currently gaining momentum as powerful methodologies for accessing functional molecular and supramolecular systems.^1^ The development of dynamic covalent synthesis (DCS) is particularly revealing in that regard. In recent years, DCS has evolved into an attractive methodology to construct complex, fully organic architectures from simple molecular building blocks linked by reversible covalent bonds. The reversibility of these covalent linkages allows for the occurrence of a self-correction mechanism that often results in the near-quantitative assembly of a single species, considered to be the thermodynamic product of the reaction. These products are thus easy to synthesize; they are robust and can be isolated and manipulated under standard laboratory conditions. Yet, the dynamic nature of the covalent linkages can be activated by specific stimuli, enabling the assemblies to respond and adapt to their environment. This activatable dynamic feature is key to the development of many molecular devices and other sophisticated systems, such as smart host–guest systems.

Historically, dynamic covalent chemistry was primarily focused on the design of building blocks able to generate nearly isoenergetic mixtures of compounds, or “dynamic covalent libraries”.^2–5^ By virtue of the reversible linkages, these libraries possess the ability to reorganise in response to external stimuli. For instance, the addition of a suitable molecular guest, can result in the amplification of the best binder.^6–10^ Since a small number of building blocks is often sufficient to generate large and diverse pools of potential candidates, these systems have proven to be particularly successful to rapidly identify new potent binders. However, generally complex libraries and low amplification factors often translate into low yields, making it challenging to use this approach as a practical synthetic tool.

Dynamic covalent synthesis has emerged from the observation that some building blocks almost exclusively assemble into a single species under specific conditions that bias the thermodynamic landscape of dynamic combinatorial libraries. What is particularly surprising is that the species formed under these conditions are often intricate and difficult to obtain through traditional synthetic methods. Interlocked species, for example, can self-assemble in near quantitative yields.^11^ Unfortunately, subtle changes in the composition and structure of the building blocks can have a profound impact on the outcome of the reaction, sometimes resulting in the formation of a single product, while other times, they lead to the formation of complex libraries. Predicting the outcome of dynamic combinatorial syntheses is further complicated by the fact that these systems are not always thermodynamically controlled. Kinetic biases have been shown to play an important role in some cases.^12,13^ Designing small building blocks able to self-assemble into specific architectures can thus be a delicate task. Nevertheless, our understanding of these types of systems has considerably improved in recent years. Correlations between the structure of the building blocks and the resulting assemblies have emerged, enabling chemists to rationally devise an increasing number of dynamic covalent syntheses. These successes are currently turning DCS into a synthetic method of choice to access particularly complex molecular architectures.

## 2. Scope of the review

**Fig. 1:**
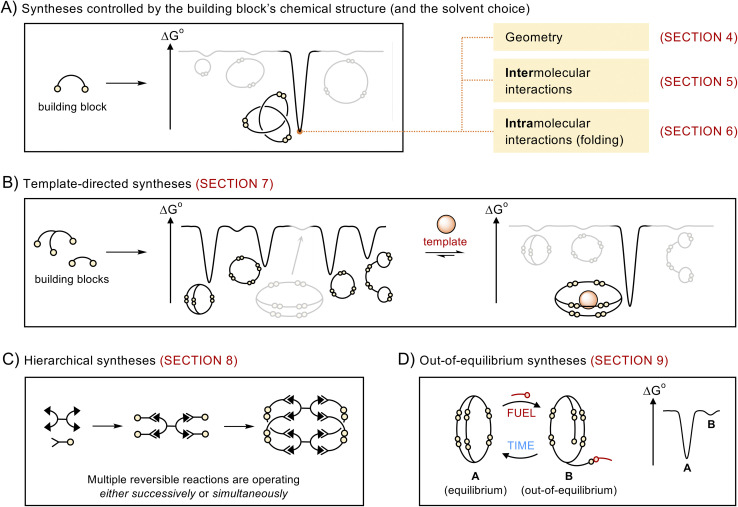
Schematic representations of the different approaches discussed in this review. Dynamic covalent synthesis can be directed by (A) encoding the relevant information in the chemical structure of the building blocks and (B) adding a suitable template. Further structural diversity becomes available using (C) hierarchical and (D) out-of-equilibrium approaches.

This article does not present a comprehensive review of all the structures obtained *via* dynamic covalent synthesis, nor does it list the large number of available dynamic covalent bonds. The aim of this review is, rather, to discuss a selected number of recent syntheses in order to outline key features that may help chemists to successfully design the future generations of building blocks and discover new types of assemblies. The different approaches discussed in this review are summarized in Fig. 1. We first describe systems that exhibit the highest levels of predictability, that is, systems in which the outcome of the reaction mostly depends on the geometry of the building blocks (Section 4). We then examine how the introduction of intermolecular (Section 5) and intramolecular (Section 6) interactions provides access to increasingly complex architectures but reduces the level of predictability in the corresponding systems. After describing the use of templates (Section 7) and multiple dynamic covalent linkages (Section 8) as a means to expand the available diversity of dynamic covalent assemblies, we explain how dynamic covalent systems can be driven out of equilibrium (Section 9). In each case, we describe the features that make dynamic covalent assemblies appealing targets for potential applications.

This review primarily focusses on the synthesis of discrete species, assembled from building blocks connected by reversible covalent bonds (*e.g.*, C=N and S–S bonds). Many of the approaches described herein can be generalised to the synthesis of discrete metal–organic assemblies and larger assemblies such as covalent organic and metal–organic frameworks. A discussion of these systems lies beyond the scope of this review, but the interested reader will be able to find relevant information in other recent reviews where these topics have been extensively covered.^14–21^

## 3. General considerations and building blocks design

Dynamic covalent syntheses are usually conducted in relatively dilute conditions (*e.g.*, in the low millimolar concentration range) to prevent unwanted polymerization and favour the formation of closed macrocyclic structures. Open structures with unreacted ends are generally not observed in appreciable quantities as the formation of dynamic covalent linkages between the building blocks is enthalpically favoured.

**Fig. 2:**
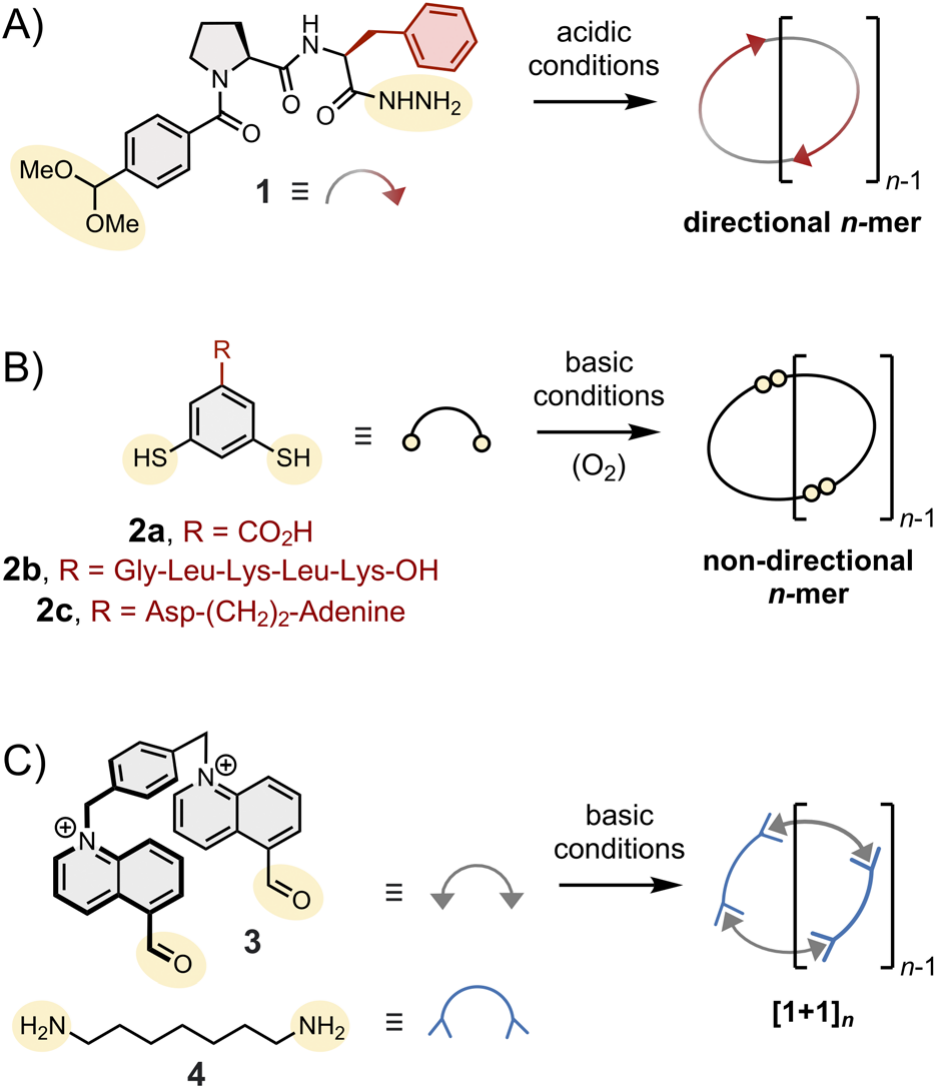
Chemical structure of typical dipodal building blocks,^11,23,25,26^ highlighting the main body in grey, the anchor for reversible linkage in yellow and side chains (if any) in red. The reversible linkages depicted in this figure are amongst the most widely used in dynamic covalent synthesis: (A) acylhydrazone, (B) disulphide and (C) imine bonds.

Fig. 2 shows selected examples of typical building blocks. These building blocks are constituted of three parts: the main body, the anchor for dynamic covalent linkage and side chains. The chemical composition of each of these parts must be carefully considered before conducting any dynamic covalent synthesis. Indeed, building blocks that have not been appropriately designed can yield disappointingly simple assemblies. Many dithiol building blocks, for example, only form closed disulphide monomers and dimers. It is tempting to infer that entropic factors always favour the assembly of the smallest possible assemblies. This is not necessarily the case, however, because the entropic penalty associated with the formation of larger assemblies can be outweighed by other factors such as: (1) the enthalpic contributions arising from the occurrence of non-covalent interactions within the larger assemblies and/or with a template; and (2) the enthalpic and entropic contributions arising from desolvation. The effect of desolvation is particularly important when the dynamic covalent synthesis is carried out in water, where the hydrophobic effect can drive the assembly of spectacularly large assemblies. In these cases, smaller assemblies cannot satisfactorily fold (or aggregate) to minimize their hydrophobic surface. Their formation increases the overall free energy of the system rather than decreasing it, which has for consequence to funnel the systems towards more complex assemblies. In practice, the role played by the different enthalpic and entropic contributions in the assembly process is subtle and difficult to evaluate. Yet, simple considerations on building block design can already provide a good level of control over the assembly in many cases.

### 3.1. Building block’s main body

The main body is arguably the most important part of a building block because it carries most of the chemical information that determines its assembly into a specific product. This information also largely determines the functionality of the resulting product.

As a rule of thumb, it is advisable to construct the main body from components that are rigid and/or possess the ability to participate in non-covalent interactions. These features direct the assembly by imposing steric constraints, favouring pre-organisation, and reducing the available conformational space. Rigid aromatic moieties are a privileged scaffold for constructing building blocks that behave in predictable ways. Amino acids and peptides are also commonly used.^22^ Despite their flexibility, they can display conformational preferences depending on their composition/sequence and depending on the solvent used. Amino acids are chiral and can partake in a broad range of supramolecular interactions, such as hydrogen bonding, electrostatic, π–π, cation–π and anion–π interactions, which make their self-assembly less predictable but particularly interesting. Highly flexible components, such as long aliphatic chains, are less commonly used because they provide limited control over the assembly. Yet, even gauche interactions (3.9 kJ mol^−1^ per interaction) can induce conformational preferences that destabilize some products and favour others.^23,24^ The addition or the removal of a single –CH_2_– from an aliphatic chain can have a profound impact on the outcome of the reactions and result in the occurrence of impressive odd-even effects.

### 3.2. Dynamic covalent linkage

The choice of the dynamic covalent linkage provides an additional level of control over the synthesis. This choice has particularly important implications on the following:

(1) The experimental conditions in which the synthesis will be performed. For example, disulphide bonds are stable in acidic conditions and become reversible in the presence of thiolates formed in basic conditions. Conversely, (acyl)hydrazone bonds are reversible in acidic conditions and stable in basic conditions. Of course, the choice of the dynamic covalent linkage also impacts the conditions that activate the assembly responsiveness.

(2) The type of assembly formed. Monopodal building blocks can only form linear dimers. Dipodal building blocks (such as those depicted in Fig. 2) form either macrocycles or linear polymers. Finally, higher order multimodal building blocks are needed to access cages.

(3) The symmetry and directionality of the assemblies formed, as illustrated in Fig. 2: hydrazone-based macrocycles (1)_*n*_ are directional,^11^ disulphide-based macrocycles (2)_*n*_ are not, and the condensation of bisaldehyde 3 and bishydrazide 4 necessarily yield macrocycles (3·4)_*n*_ with alternating subunits.

(4) The conformational constraints imposed on the assemblies. For instance, the dihedral angle of the disulphide C–S–S–C bond is around 90°. Imine and (acyl)hydrazone bonds are planar in their fully relaxed states but tolerate significant twisting.^27,28^

### 3.3. Side chains

Side chains are often introduced to increase solubility, but their role in the assembly is not always innocent and should not be underestimated. The behaviour of building block 2 has been shown to be particularly sensitive to its side chain composition. Without side chains, 2a mostly forms a mixture of 3mer and 4mer in aqueous media.^26^ The introduction of the short peptide side chain -Gly-Leu-Lys-Leu-Lys-OH (2b) promotes strong intermolecular interactions between 6mers (and between 7mers), which become amplified as a result of self-replication. In contrast, the introduction of a side chain combining aspartic acid and adenine (2c) promotes the formation of an unusually large 15mer *via* intramolecular folding!^29^ The important role of these side chains is further discussed in Section 6.

In the next sections, we analyse how the above-described features translate into the assembly of different types of structures.

## 4. Systems dominated by geometry

**Fig. 3:**
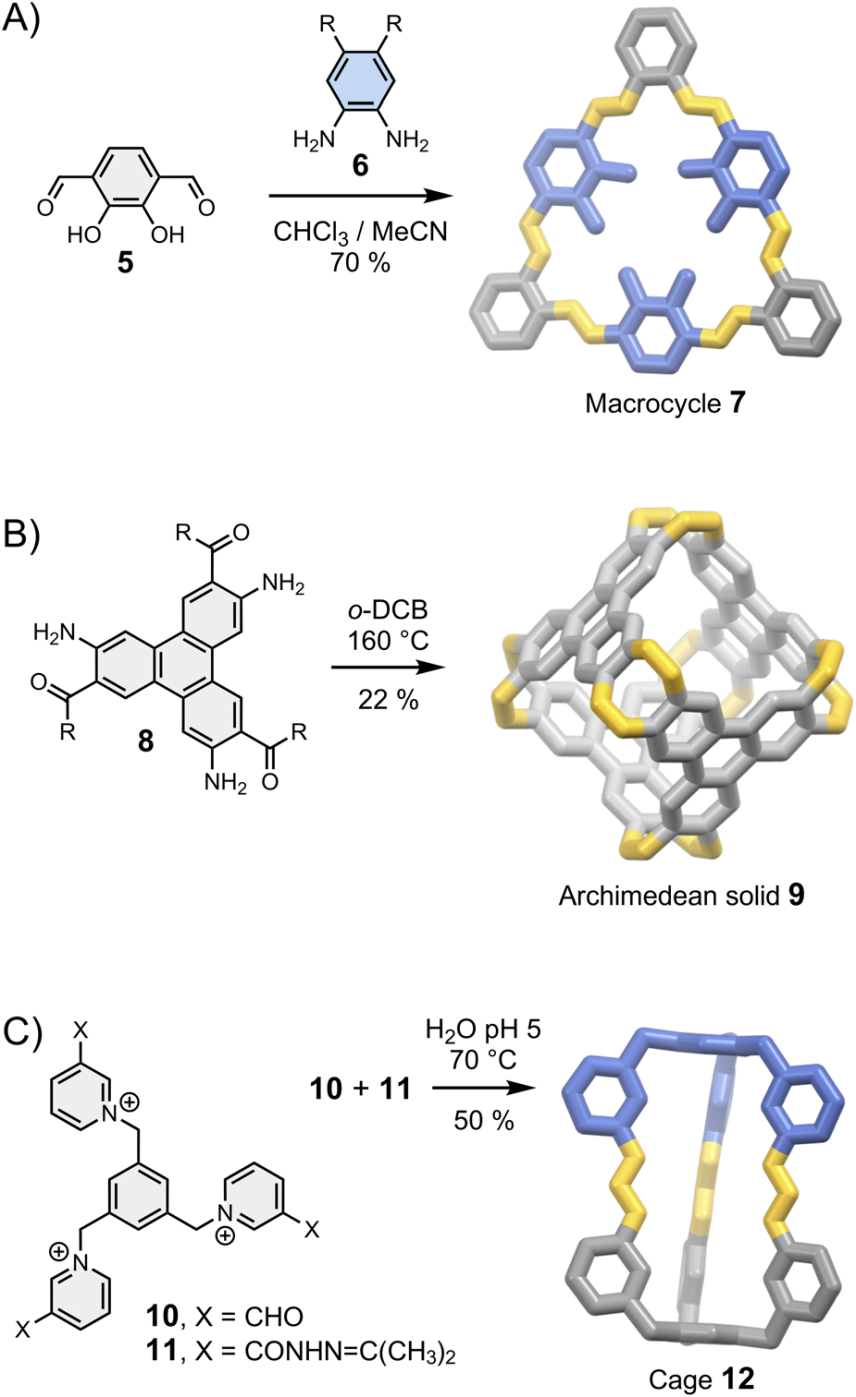
Assembly of (A) a macrocycle (MacLachan),^32^ (B) an Archimedean solid (Wu)^27^ and (C) a cage (Li)^33^ controlled by the geometry of the building blocks. Side chains R are omitted for clarity.

Let us first consider the simplest case scenario in which the geometrical features of a building block only allow for the formation of a single assembly: the building blocks just “click” together with the minimum involvement of any supramolecular interactions, just like the pieces of a three-dimensional puzzle (Fig. 3). The corresponding syntheses are generally efficient (>80% yield per new bond formed for the assemblies shown in Fig. 3) because of the favourable orientation of the reactive sites. After the first connection, the formation of the other chemical bonds becomes intramolecular, which leads to positive cooperativity funnelling the thermodynamic landscape toward the final product. This situation is generally encountered when the resulting assembly is sufficiently constrained to preclude the occurrence of any intramolecular interaction that may bias the equilibria or lead to mixtures of conformational/configurational isomers in equilibrium. In this case, the outcome of the reaction mostly depends on the geometrical constraints imposed by the building block's structure. This simple consideration provides the basis for the most reliable strategy used to control the assembly of fully organic architectures. Indeed, the relationship between the geometry of a building block and the assembly of two- and three-dimensional products is well understood and benefits from decades of related work on the self-assembly of metallo-macrocycles and cages pioneered by Fujita, Stang and others in the 1990s.^30,31^

In brief, rigid dipodal building blocks with matching geometries form shape-persistent macrocycles. This behaviour is exemplified in Fig. 3A with the assembly of imine-based macrocycle 7 reported by MacLachan.^32^ The synthesis of 7 clearly depends on the geometry of building blocks 5 and 6: the type and the size of the product formed is determined by the distance and the angle separating reactive groups placed around a rigid aromatic body. It is interesting to point out that macrocycle 7 can self-associates *via* face-to-face π–π interactions to form tubular aggregates but this phenomenon, which provides the basis for the self-assembly of supramolecular polymers,^1^ does not influence the outcome of the synthesis. Many other types of shape-persistent macrocycles can be obtained using a geometry-based approach, including cyclophanes^34^ and *m*-phenylethylene macrocycles.^35^

Higher order multipodal building blocks can be designed to yield cages with well-defined cavities suitable for host–guest chemistry, and the use of fully organic linkages provides opportunities for new properties unavailable with metal-coordination cages. The Archimedean solid 9 reported by Wu *et al.*^27^ in 2021 (Fig. 3B) nicely illustrates this statement: linking aromatic building blocks 8 with imine bonds extends the π-conjugated systems to the entire structure, opening a new route towards fully fused π-conjugated cages.

In some cases, the directional nature of imine or (acyl)hydrazone bonds can be exploited to obtain dissymmetric cages with ease. Li *et al.* have reported a particularly striking example with the assembly of cage 12 from complementary building blocks 10 and 11 (Fig. 3C).^33^ Dissymmetry is an appealing feature from the perspective of function because it inherently increases structural complexity. The cavity of dissymmetric cages can be notably used to encapsulate chiral guests and form unconventional host–guest complexes. In the above-mentioned example, cage 12 possesses two inequivalent binding sites within a single cavity, enabling the recognition of heteroanion dimers.^33^

Last but not least, the presence of dynamic covalent bonds imparts chemical responsiveness to the assemblies, allowing control over guest release as a function of physical triggers (temperature, concentration) and chemical triggers (pH, reactive exchange partners).^36^ This property is particularly appealing in the contexts of drug delivery and wastewater decontamination where active release and cycling processes are respectively needed. Recent years have thus witnessed a surge of interest in the dynamic covalent synthesis of water-soluble molecular containers. Li *et al.* have described the use of reversible imine, hydrazone and oxime condensation to synthesize water-soluble macrocycles and cages that display remarkably high affinities for hydrophobic aromatic guests and anions in pure water.^37–39^ Recent work by White *et al.* has revealed that this type of structures can even sequester sulfate,^40^ an anion notoriously difficult to capture in water because of its high solvation energy. The guests can be released when the dynamic bonds either break, shuffle, or isomerize. The pH-dependent NH deprotonation of (acyl)hydrazone-based macrocycles, for example, triggers a conformational isomerization that changes the geometry, the π-electron density, and the number of available hydrogen-bond donors of the entire structure, thereby altering its recognition properties.^41,42^

## 5. Syntheses driven by non-covalent intermolecular interactions

**Fig. 4:**
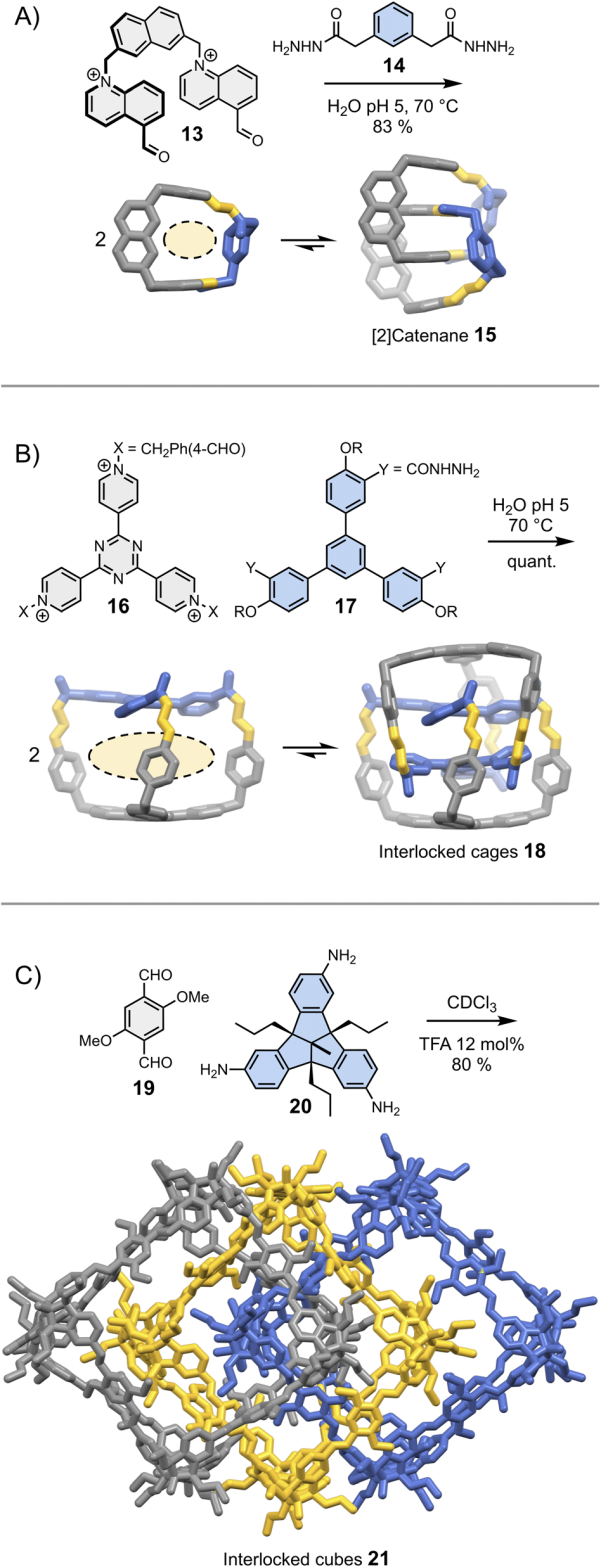
Dynamic covalent synthesis of (A) a [2]catenane (Cougnon),^44^ (B) triply interlocked cages (Li & Sessler)^45^ and (C) triply interlocked cubes (Mastalerz).^46^ Side chains R are omitted for clarity.

In most cases, the geometric features of a building block allow for the formation of multiple assemblies. When several assemblies are possible, there are two situations in which intermolecular interactions may drive the assembly of a single product. In the first situation, one of the assemblies self-associates more strongly than the others and becomes amplified as a result of self-replication.^26^ Self-replicating systems and their ability to mimic the behaviour of living systems have been recently reviewed elsewhere.^43^ In the second situation, one of the assemblies self-associates to form a thermodynamically stable (and kinetically inert) mechanically interlocked molecule, following an approach illustrated by a recent report of Cougnon *et al.* (Fig. 4A).^44^ The condensation of 13 and 14 in water yields a rigid [1 + 1] macrocycle with relatively large hydrophobic cavity and apertures. This macrocycle resembles an open box. Steric constraints prevent both its folding and the occurrence of intramolecular interactions between the aromatic units. Since both the cavity and the apertures are quite large, another building block can thread in the hydrophobic cavity, ultimately leading to the formation of [2]catenane 15. The formation of this [2]catenane is somewhat analogous to bimolecular aggregation. It is driven by the synergistic effects of π-stacking and the need to release high energy water molecules located in the macrocycles' hydrophobic cavity. This approach is generalizable to more complex structures, and has been notably employed by Sessler and Li to obtain triply interlocked cages 18 from the condensation of 16 and 17 (Fig. 4B).^45^

These two examples were selected because they demonstrate that simple designs involving a minimum number of non-covalent interactions can already provide access to great structural complexity with a reasonable level of control. It is important to keep in mind that seemingly insignificant building block features can have unpredictable – but major – consequences on the assembly. An example of such unexpected behaviour was reported by the Mastalerz group in a 2022 study.^46^ The dynamic covalent synthesis of interlocked imine cubes 21 in chloroform starting from bisaldehyde 19 and trisamine 20 (Fig. 4C), is driven by Keesom and London dispersion interactions between the methoxy side chains and with the solvent, rather than by π-stacking. Replacing the methoxy side chains by hydroxyls thus disrupts the formation of 21, leading to the formation of the simpler, non-interlocked monomeric cubes. Evidently, this dramatic effect could not have been predicted.

It is interesting to note that the formation of all the interlocked structures depicted in Fig. 4 breaks the symmetry of the individual components (*i.e.*, macrocycle, cage or cube), resulting in the emergence of chirality. With their large size, comparable to that of peptides and small proteins, enantiomerically pure interlocked assemblies would certainly find important uses in recognition and catalysis. Unfortunately, both the stereoselective synthesis of such complex structures and the separation of the different stereoisomers are currently challenging.

## 6. Syntheses driven by non-covalent intramolecular interactions (or folding)

Intramolecular interactions can induce the folding of a specific assembly, stabilizing it relative to competing macrocycles that are unable to engage in such interactions. In these cases, the reaction yields the most efficiently folded structure.

### 6.1. Synthesis of folded oligomers

**Fig. 5:**
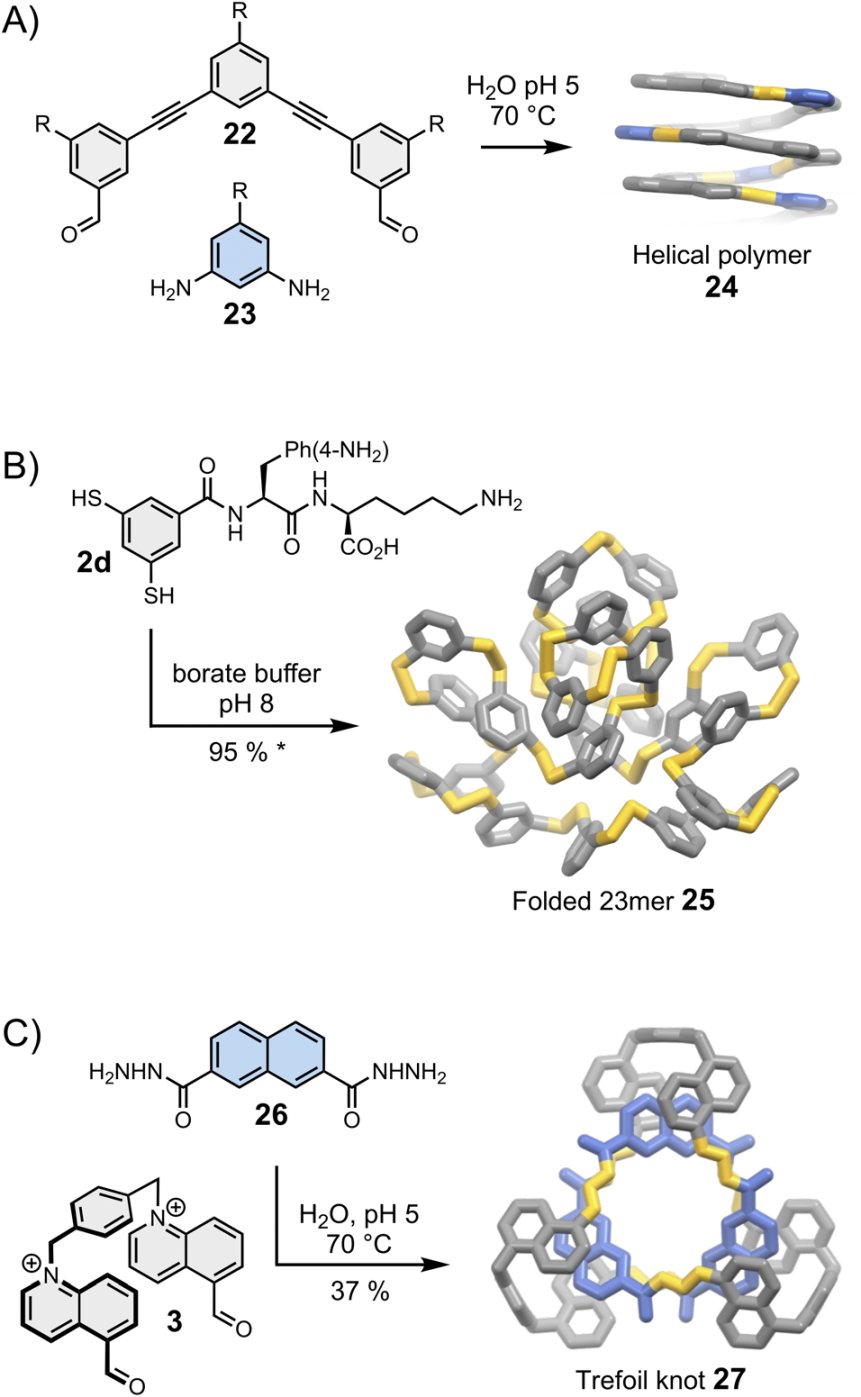
Folding-driven assembly of (A) helical polymers (Stupp),^51^ (B) an unusually large folded 23meric macrocycle (Otto),^25^ and (C) a trefoil knot (Cougnon).^53^ Side chains “R” are omitted for clarity. The yield marked with a star was measured by HPLC.

Moore was arguably the first one to recognise the importance of folding in dynamic covalent syntheses.^47,48^ His group had previously established the solvophobicly-driven folding of oligo(*m*-phenyleneethynylene), or oligo(*m*-PE).^49^ Realizing that imine and acetylene bonds have commensurate geometry, they investigated whether an imine linkage could be used to connect two oligo(*m*-PE) building blocks and discovered that the reversible condensation was controlled by the oligomer folding.^48^ Since then, this approach has been revisited and simplified by Lehn *et al.*^50^ and Stupp *et al.* (Fig. 5A).^51^ The oligo(*m*-PE) scaffold is particularly rigid, and its folding obeys predictable geometric rules. We have previously mentioned that oligo(*m*-PE) building blocks with matching geometries form closed macrocycles^35^ (Section 4). In Stupp's strategy, a geometrical mismatch between building blocks 22 and 23 prevents the formation of a small, closed macrocycle, favouring the production of amphiphilic polymers 24 that adopt a helical conformation in water to minimize the hydrophobic surface exposed to the solvent.

### 6.2. Synthesis of folded macrocycles

Harnessing folding to direct the dynamic covalent synthesis of closed monodisperse assemblies, such as macrocycles, is significantly more challenging. Macrocycles can fold only if they possess a certain level of flexibility. Unfortunately, the presence of a small number of flexible components considerably increases the available conformational space, thereby reducing the chances to accurately predict the most stable folded state.

The assembly of stable folded macrocycles can be promoted by introducing biomolecular moieties into building blocks that display well-established folding propensity. The Otto group has reported particularly remarkable examples of folding-driven dynamic covalent syntheses following this approach. A typical example of their work in this field in shown in Fig. 5B. In an earlier example, the group had reported the spontaneous assembly of a large folded 15meric macrocycle^52^ from chimeric building block 2c (previously shown in Fig. 2B), composed of a rigid aromatic moiety decorated with aspartic acid and adenine. Amino acids and nucleobases are key structural elements involved in the folding of proteins and nucleic acids, but they are rarely combined within the same building block. Here this combination results in the formation of a macrocycle that displays fundamentally new folded secondary and tertiary motifs, unlike the commonly observed helix and sheet motifs dominating in natural biopolymers. Smaller assemblies do not form in significant proportion because they cannot fold to shield their hydrophobic parts from water and maximize the number of favourable interactions between the side chains.

The group later realised that other side chains, which do not blend amino acids and nucleobases, could also bias the equilibria towards unusually large – but perfectly folded macrocycles. Side chains combining natural and unnatural amino acids, for example, lead to the assembly of multiple low symmetry, unconventional foldamers composed of 13, 17 and 23 repeating units.^25^ The use of lysine and *p*-NH_2_ substituted phenylalanine (building block 2d, Fig. 5B) exclusively yields the corresponding 23mer (25). Interestingly, minor changes in the experimental conditions tilt the delicate balance of interactions involved in the assembly, destabilizing the folded 23mer and favouring the formation of a 6mer that self-replicates *via* aggregation.^54^ Replacing the *p*-NH_2_ substituted phenylalanine by a *p*-CO_2_H substituted phenylalanine even allows the pH-dependent, reversible transformation between folded 16meric and 9meric macrocycles.^55^ The idea to exploit the presence of side chains to bias the equilibria in favour of the most stable assembly is relatively recent but these first examples unambiguously demonstrate the great potential of this approach to direct the synthesis of large folded macrocycles.

An alternative approach to create folded monodisperse macromolecules simply consists in using pre-folded building blocks. Along these lines, the Link group has recently described the transformation of a naturally folded lasso peptide into a mechanically-interlocked building block suitable for the generation of disulphide-based libraries of rotaxane-like compounds.^56^ This strategy provides access to a new family of mechanically-interlocked peptides, which had been difficult to achieve until then. However, it does not exploit the full potential of dynamic combinatorial synthesis, as the disulphide bond is merely used as a connector and its reversible nature is not used to explore regions of the thermodynamic landscape that would not be accessible otherwise, as was done in the previous examples.

### 6.3. Synthesis of molecular knots

Folding can also drive the assembly of particularly compact knots and multi-entangled macromolecules (Fig. 5C). Sanders was the first to report, in the early 2010s, the dynamic covalent synthesis of a trefoil knot^57^ and other entangled macrocycles^58^ from building blocks composed of naphthalenediimide moieties connected by amino acids. The syntheses are performed in water and folding is driven by the need to minimize the hydrophobic surface area of the naphthalenediimide moieties in contact with the aqueous environment. Moreover, the presence of chiral centres in the building blocks induces the handedness of the entangled macrocycles. While these discoveries were purely fortuitous, the Cougnon group has now demonstrated that dynamic covalent systems can be intentionally designed to produce entangled structures. Even simple amphiphilic building blocks (3 and 26, Fig. 5C) can assemble into complex structures like trefoil knot 27 in water.^59^ The exact topology of the macrocycles formed in the course of these reactions remains, however, impossible to predict. The geometry of the individual building blocks was found to be particularly critical, and the syntheses work well only if all the building blocks perfectly fit together. This work is currently under further development but the recent report of the assembly of another trefoil knot from a biphenylene-based bisaldehyde and (1*S*,2*S*)-(+)-1,2-diaminocyclohexane by Li *et al.*^60^ hints that solvophobic effects may provide easy access to functionally diverse entangled macrocycles.

Before closing this section, we must reiterate that folding represents an extremely promising tool to access new, highly sophisticated three-dimensional molecular architectures but is particularly difficult to harness. So far, a limited number of new folds has been produced. Folding has mostly been applied to direct the assembly of open polymers and macrocycles. The folding-driven assembly of other types of structures (*e.g.*, folded cages) remains to be explored, although first steps have already been taken in that direction. It has been shown, for example, that directing groups partaking in intramolecular interactions (such as hydrogen bonds) can influence the formation of dynamic covalent cages.^61,62^ It is clear that the current studies are only scratching the tip of the iceberg, and folding-driven syntheses will surely develop in new directions in the coming years.

## 7. Syntheses based on the use of templates

In the examples described until now, the product is spontaneously and autonomously produced through a programmed assembly. Indeed, the information required to control the assembly is encoded only in the molecular structure of the organic building block(s) and in the type of connectivity chosen. The use of templates provides an additional layer of complexity. Templates primarily, but not exclusively, affect chemical processes *via* non-covalent interactions such as hydrogen bonding, π-stacking, or the hydrophobic effect. The most common templates are metal ions. Here we exclusively consider them as templating agents even though coordinate bonds, also known as dative bonds, are often reversible and may also be considered as another type of dynamic covalent bonds. Templates guide the reaction pathway towards the preferential formation of a particular target from several reactants that in principle have the potential to react or assemble in many different ways.^63^ Templates can thus be effectively applied to direct the synthesis of highly complex products with particular geometries and spatial arrangements.^64^

### 7.1. The template effect

The strategic role of templates has been recognized and successfully applied to countless chemical processes. Templates are also ubiquitous in nature. Perhaps the most important example is DNA replication, where a single strand of DNA templates the formation of a complementary strand through the assembly of nucleotide bases. In 1956, Todd proposed the idea that templates, like those found in nature, could eventually be employed in the laboratory to govern synthesis, mirroring the distinctive selectivity characteristic of biological templates.^65^ In retrospect, it seems that synthesis facilitated by metal-ion templating was accomplished as far back as 1926, when Seidel obtained the macrocycle from 2-aminobenzaldehyde and ZnCl_2_.^66^ It was through the ground-breaking efforts of Busch during the 1960s that the significance of templates in synthesis became elucidated and advanced.^67–69^ Busch provided the following definition for the template function: “A chemical template organizes an assembly of atoms with respect to one or more geometric loci, in order to achieve a particular linking of atoms”. In fact, he was the first to intentionally use a metal ion template in the synthesis of the bis-imine macrocycle from the reaction of a nickel(ii) dithiolate complex with 1,2-bis(bromomethyl)benzene (in the absence of the Ni(ii) template other cyclic and acyclic products were formed).^70,71^ Since this time, a wide range of structurally and geometrically intricate architectures such as macrocycles, cages, catenanes or knots have been efficiently synthesized by relying on the assistance of inorganic or organic templates. Control of molecular topology has remained a significant focus within a field of template-directed synthesis ever since Sauvage *et al.* accomplished in 1983 the first efficient synthesis of a [2]catenane, through the use of coordination bonds between phenanthroline-based ligands and a tetrahedral Cu(i) centre, a commonly employed template type in the construction of intricate architectures.^72^

Lehn *et al.* provided a seminal demonstration of the importance of the template effect in dynamic combinatorial systems when reporting the quantitative anion driven synthesis of pentameric and hexameric circular helicates from equimolar amounts of tris-bipyridine ligands and iron(ii) chloride and sulfate, respectively.^73,74^ The spontaneous and exclusive generation of these highly complex assemblies is only possible in the presence of templating anions, which engage in electrostatic interactions with the cationic complex and fit perfectly into the inner cavity of the cyclic helicates. Significantly, each helicate exhibits high selectivity toward a particular anion, and the use of anions with other sizes and geometries led to the formation of either a mixture of products or undefined metallo-supramolecular species. Templates are not necessarily ionic, and the Anderson group has provided striking examples of the use of neutral organic templates in the synthesis of π-conjugated porphyrin nanorings and nanoballs of various size.^75^ The combination of linearly coordinating metalloporphyrin (acting as reactants) and oligopyridine ligands (acting as templates) allows a variety of porphyrin-based arrays to be created with very high selectivity.^76,77^ Again, the use of suitable templates provides access to structurally complex products that would otherwise remain inaccessible.

### 7.2. Templated dynamic combinatorial synthesis of macrocycles

The template effect can also be harnessed to direct dynamic covalent syntheses. Template-directed DCS operates on two levels of dynamics, comprising reversible covalent linkages between individual building blocks and non-covalent interactions (although, covalent template effects are also known)^81,82^ induced by the presence of template. The synergistic and often simultaneous interplay of both types of reversibility within a single system provides a pathway towards complex products, whose composition may be subsequently altered by the application of external physical or chemical stimuli.

**Fig. 6:**
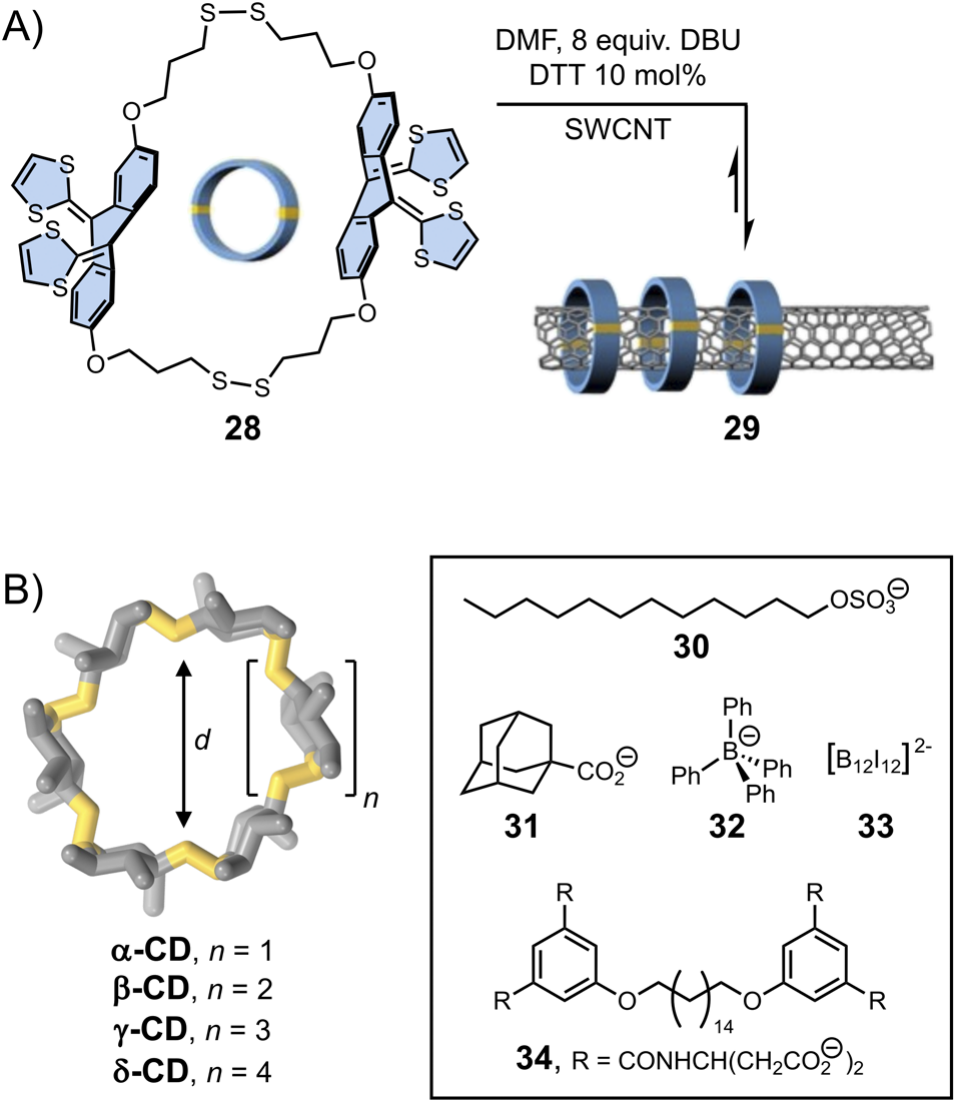
(A) Single-walled carbon nanotubes (SWCNT) functionalization with dynamic disulphide macrocycles (von Delius). Adapted with permission from ref. 78 (copyright 2020 John Wiley and Sons); (B) template-directed synthesis of cyclodextrins with different cavity sizes (Beeren).^79,80^

Template-directed DCS was first applied to the synthesis of macrocycles. The yield in macrocycle synthesis is often low owing to the competition between ring closure and oligomerization. In the early 2000s, Otto and Sanders presented an aqueous dynamic library of disulphide-based macrocycles generated by three simple dithiol organic building blocks.^83^ Exposure of this library to templates (2-methylisoquinolinium iodide or *N*-methylated morphine) caused a dramatic change in the library composition. Addition of the latter resulted in the quantitative formation of a single macrocyclic product (*via* cation–π interactions with the template) – a structure essentially unobserved in the non-templated library. More recently, von Delius *et al.*^78^ showed that disulphide exchange could also be applied to promote the formation of dynamic macrocycles 28 based on rigid and curved π-extended tetrathiafulvalene-motif around one-dimensional single-walled carbon nanotubes (SWCNT) acting as a template (29, Fig. 6A). This work elegantly exploits the building blocks features (in terms of shape, electronic and structural features) and the action of a suitable template to stimulate the error-correction mechanism during the formation of the interlocked dynamic disulphide macrocycles.

The formation of larger cyclic architectures *via* template-assisted synthesis remains challenging, with some notable exceptions.^84–86^ Li *et al.* have reported a system in which the reversible condensation of an aromatic dihydrazide and a pyridinium-based bisaldehyde in water yields a mixture of small [1 + 1] macrocycle and [2]catenane in the absence of any template. Depending on the composition of the bisaldehyde building block, the equilibria can be shifted towards either a [2]pseudorotaxane or an ultra-large [2 + 2] macrocycle upon addition of an external template, namely cucurbit[8]uril, which forms ring-in-rings complexes *via* aromatic donor–acceptor interactions with the pyridinium units. Template-directed dynamic covalent synthesis can also provide access to unusually large cyclodextrins (CDs) that are only formed in trace amounts during the industrial production of these compounds. The Beeren group has developed the use of enzyme catalysis to generate dynamic mixtures of interconverting cyclodextrins, which become stable after denaturation of the enzyme.^80^ They have demonstrated that the addition of hydrophobic guests of different sizes (30–33, Fig. 6B) promotes the highly selective amplification of α-, β-, γ- and δ-CDs, respectively, sometimes resulting in the near quantitative formation of one type of cyclodextrin. In a recent example,^79^ the group has showed that the use of bolaamphiphile templates (such as 34) enables the efficient synthesis of large-ring δ-CD, *via* the formation of a [3]-pseudorotaxane. Using an optimised protocol, δ-CD could be isolated with an unprecedented 7.2% yield (against 0.2% in previous reports).

**Fig. 7:**
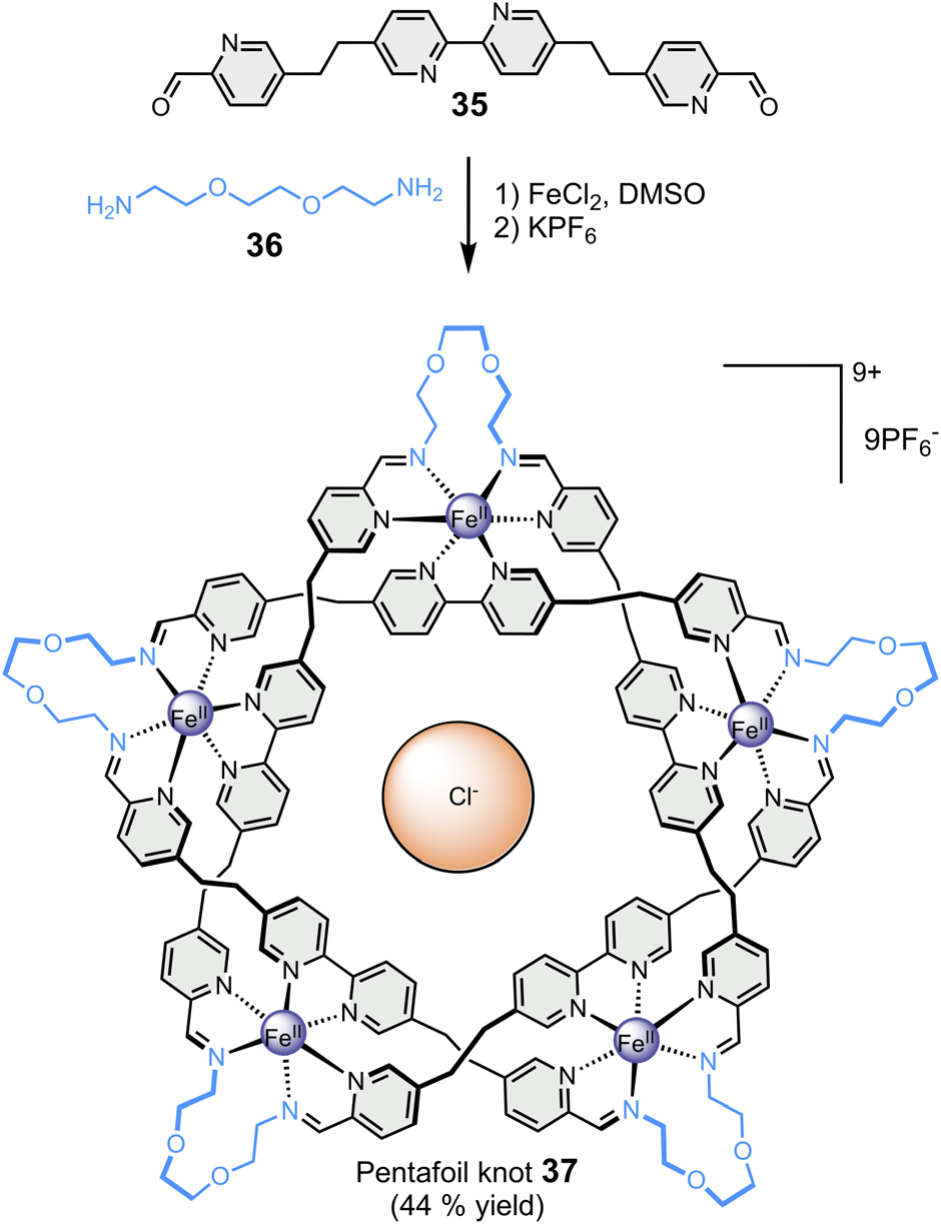
Template-directed synthesis of a pentafoil knot (Leigh).^87^

Finally, the Leigh group has reported the particularly remarkable synthesis of an imine-based synthetic molecular knot (Fig. 7), achieved through the application of the template effect.^87^ In a one-step reaction, the self-assembly of five bis-aldehyde 35 and five bis-amine 36 around five metal cations, namely Fe(ii), and one chloride anion led to the formation of 160-atom-loop molecular pentafoil knot 37. The synthesis of this highly complex architecture would not have been possible without the templating role of the centrally located chloride anion, which welded all the components together through a network of ten CH⋯Cl^−^ hydrogen bonds.

### 7.3. Templated dynamic combinatorial synthesis of cages

**Fig. 8:**
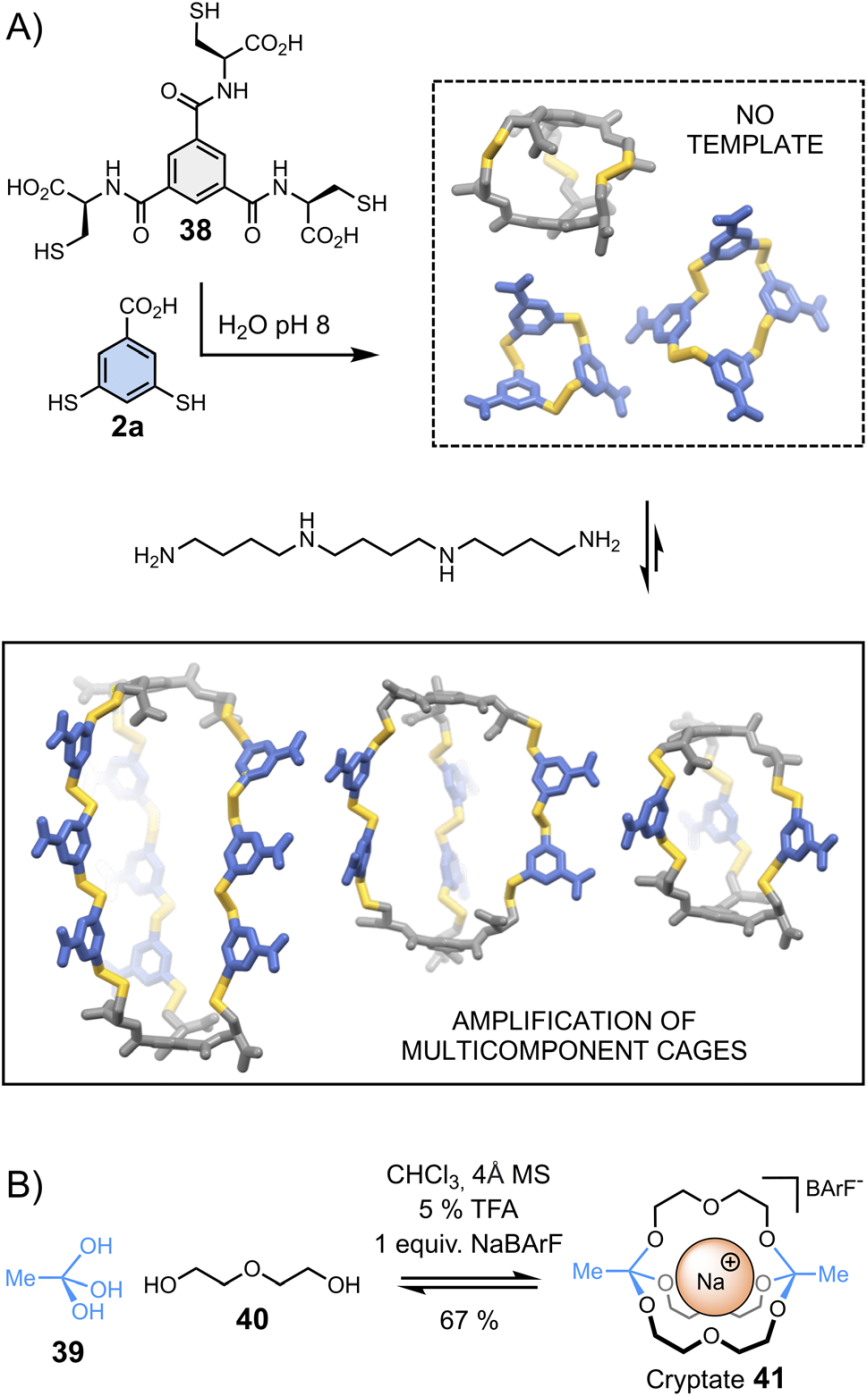
Template-directed synthesis of (A) multicomponent disulphide cages in water (Stefankiewicz)^97^ and (B) cryptates (von Delius).^100^

Cages represent another class of complex architectures with high application potential whose construction is not trivial, especially when they are composed of more than two components.^88–96^ One proposed methodology for synthesizing multicomponent cages involves the dynamic covalent connection of di- and trifunctional building blocks. Stefankiewicz *et al.*^97^ have applied this approach to generate a new class of multi-component disulphide cages in water (Fig. 8A). These unusual architectures, assembled from simple tri- (38) and dithiol (2a) building blocks at near physiological pH, can contain up to eleven building blocks. The formation of multicomponent cages occurs exclusively when templated by an appropriate polyamine, which interacts *via* electrostatic interactions with the building blocks' carboxylates, directing the generation of the desired cages.

Combinations of different templates have been employed by von Delius *et al.*^98–100^ to synthesize macrobicyclic cages 41 – or cryptates – based on the urea/triethylene glycol binding motifs 40 and dynamic orthoester bridgeheads 39 (Fig. 8B). In early examples, the reversible covalent synthesis of triethylene glycol-based cryptates was driven by the addition of alkali metals (Li^+^, Na^+^, K^+^, Cs^+^) – in the absence of template, an eight-membered macrocyclic structure was formed. More recently, the same group adopted a similar approach to obtain orthoester cages comprising urea moieties, which bind anionic templates (Cl^−^, Br^−^, I^−^ and NO_3_^−^) and ion pairs (CsCl).

Chiral cages can also be synthesized by application of a template. Ballester *et al.*^101^ reported the templated self-assembly of dynamic imine capsules based on the condensation of a calix[4]pyrrole-tetraaldehyde and 1,2-substituted chiral diamines. An octaimine cage was quantitatively assembled as a single diastereomer in the presence of a cationic guest (bispyridyl-*N*-oxide derivatives). The template, confined within the polar interior of the cage, arranges the formyl groups of the neighbouring calix[4]pyrrole hemispheres in a configuration well-suited for the pairwise imine formation reactions involving diamine linkers.

These selected examples represent only a small part of what is known and described in the literature, especially in the context of the metal templated synthesis of functional macrocyclic and cage-like architectures *via e.g.* reversible boronic ester or imines bonds.^102–107^ Templates serve as guiding frameworks, facilitating the controlled assembly of molecular components into specific arrangements that cannot be achieved through conventional synthetic methods. The strategic use of templates has the potential to unlock new realms in molecular design. It also provides access to dynamic covalent systems able to re-organise upon template addition and removal, a desirable property in the design of new responsive materials and advanced molecular machinery.

## 8. Syntheses based on multiple reversible covalent reactions

### 8.1. Combining multiple reversible covalent reactions

An obvious goal of dynamic covalent synthesis is to move away from assemblies composed of only one or two building blocks to build complex multi-component assemblies. Combining multiple reversible covalent reactions within a single system allows for the hierarchical assembly of complex organic structures in one-pot.^108,109^ In contrast with traditional domino/cascade reactions, which are essentially irreversible,^110,111^ a dynamic covalent approach should enable several self-correction and/or self-sorting processes to operate synergistically, thus funnelling the synthesis toward a single thermodynamic product.^97,112,113^ This approach was recently applied to the preparation of multi-component cages, which are key targets of supramolecular chemistry.^20,114–116^

Combining successively different reversible covalent transformations represents the simplest way to alleviate the need for orthogonal chemo-selectivity. This approach requires carrying out several reactions one after the other, increasing reaction times and complicating procedures. A more appealing approach consists in developing reversible covalent reactions that operate both orthogonally (*i.e.*, chemo-selectively) and simultaneously. Several reversible covalent reactions have been reported to operate by combination of two (imine/olefin metathesis,^117^ imine/boronic ester,^20,107,118,119^ B–N and B–O bonds within amidoboronates^120^), three (disulphide/hydrazone/boronic^121,122^ and disulphide/imine/boronic^123^), and even four coupling reactions (thiol addition/hydrazone/boronic ester/coordination^124^).

The most popular reversible covalent reactions used in dynamic covalent chemistry are undoubtedly the acylhydrazone and disulphide reactions. These chemistries are usually considered to be perfectly orthogonal and operating in different conditions: acidic pH for acylhydrazones *vs.* basic pH for disulphides.^125–127^ This orthogonality has notably enabled the Leigh group to develop molecular walkers,^128^ wherein attachment of each feet to the track involves reversible covalent connections operating step by step under different reaction conditions – the remaining foot locked onto the track preventing spontaneous release of the walker and random motion. Acylhydrazone and disulphide chemistries are not always orthogonal. The Otto group has discovered the existence of an intermediate pH range where the two reversible covalent reactions simultaneously occur. Organocatalysts that facilitate hydrazone exchange can be used to extend the reaction conditions where both reactions operate simultaneously.^127^ Alfonso has shown that the presence of DMSO as a co-solvent in aqueous media can further promote thiol oxidation into disulphides and disulphide exchange, thereby enabling acylhydrazone and disulphide chemistries to operate simultaneously in dynamic combinatorial libraries over a wider pH range.^129^

### 8.2. Hierarchical dynamic covalent synthesis of cages and networks

**Fig. 9:**
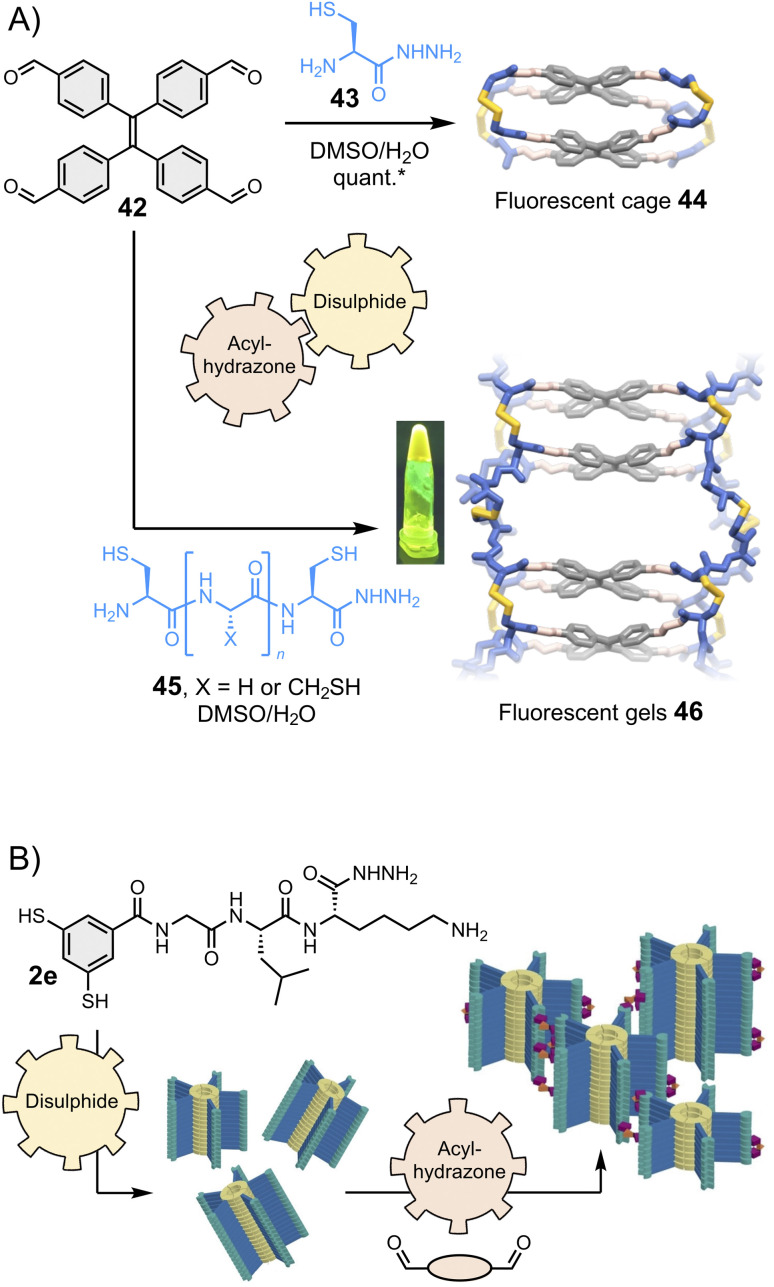
(A) Hierarchical assembly of fluorescent cages^130^ and gels,^131^ shown under UV light in the inset (Stefankiewicz and Ulrich). The yield marked with a star was measured by HPLC. (B) Hierarchical assembly of smart hydrogels (Otto), adapted with permission from ref. 132 (copyright 2023 John Wiley and Sons). All these structures are constructed using a combination of two reversible covalent reactions, namely acylhydrazone and disulphide reactions.

Stefankiewicz and Ulrich found that tetraphenylethene-based building block 42 (Fig. 9A, top) can react with heterobifunctional spacers bearing both hydrazide and thiol moieties (such as 43) to generate fluorescent organic cages displaying aggregation-induced emission through the one-pot combination of eight acylhydrazone and four disulphide bonds.^130^ Similar tetraphenylethene-based cages were recently shown to bind aromatic guests.^133–135^ The robustness of the synthetic approach enables for structural diversification. Varying the spacer composition does not alter the covalent assembly of the fluorescent cages and enables tuning of their solubility.^136^ Finally, the introduction of additional reactive thiol groups in 45 enables cross-linking, providing access to smart materials such as the fluorescent gel 46 that display multiple dynamics (Fig. 9A, bottom).^131^ The Otto group has similarly introduced a hydrazide pendant at the periphery of disulphide-based supramolecular polymers and found that hydrogels form upon combination with bis- and tris-aldehydes (Fig. 9B).^132^

### 8.3. Multi-dynamics and post-transformations

We have already emphasized that dynamic covalent assemblies display dynamic features, making them responsive and adaptable in specific conditions.^14,36,91,137,138^ For example, cages may adapt their size and shape to templating guests,^97,139^ and physico-chemical stimuli can be used to exert a control over the structural integrity of the product(s). Using several types of reversible bonds multiplies the possibilities to activate and deactivate these dynamic features. The Ulrich group achieved the controlled degradation of the multi-component fluorescent cages and gels shown in Fig. 9A using multiple chemical effectors: methoxyamine triggers acylhydrazone exchange, and β-mercaptoethanol triggers disulphide exchange.^131^ The process can be monitored by specific changes in the fluorescence and circular dichroism spectra, which correlate with different mechanisms depending on the effector. In a very recent example, Martín and co-workers reported the metamorphosis of cage compounds by component insertion *via* a dynamic covalent exchange process that is entropy-driven through solvent release.^140^

While the dynamic systems are advantageous for applications that require adaptability, freezing the product dynamics can benefit applications that require long-term stability. The Zonta group recently reported an imine reversible covalent reaction coupled to an irreversible [3,3] Diaza-Cope rearrangement, which takes place *in situ* and yields stable covalent cages.^141^ The Mastalerz group has described the coupling of an imine reversible covalent reaction with a Povarov cyclisation to obtain porous quinoline cages.^142^ The same group has also reported the Pinnick oxidative transformation of imine cages into amide cages,^143^ and even the three-step transformation of imine cages into hydrocarbon cages.^144^ Of note, it has been demonstrated that the crowded environment of interlocked cages can be exploited to control the selective transformation of the most exposed imine bonds without affecting the most hindered ones,^145^ potentially opening opportunities for further post-functionalisation.

## 9. Out-of-equilibrium dynamic covalent synthesis

**Fig. 10:**
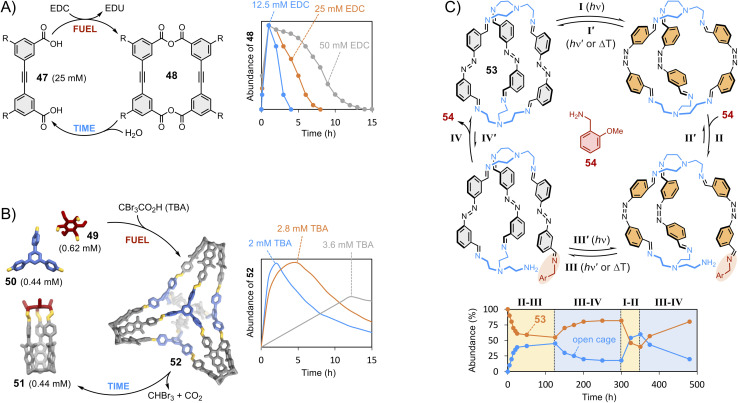
Transient synthesis trough dissipative dynamic covalent chemistry: (A) time-resolved formation of macrocycle 48 from diacid 47 as a function of the concentration of EDC fuel (Hartley);^148^ (B) time-resolved formation of covalent basket cage 52 from cage 51 and imines 49 and 50 as a function of the concentration of self-degrading tribromoacetic acid fuel (Badjić);^150^ (C) out-of-equilibrium opening of cage 53 (Feringa)^153^ triggered by photo-switching (steps I and III) followed by displacement *via* dynamic covalent exchange with amine 54 (steps II and IV). Side chains “R” are omitted for clarity.

We have now discussed the main strategies employed to control the composition of thermodynamically controlled dynamic covalent systems. To conclude, we would like to briefly introduce out-of-equilibrium systems. The growing interest of the community in out-of-equilibrium dynamic covalent systems represents a paradigm shift in the field. Supramolecular chemistry was originally focused on the synthesis of static hosts designed for a given guest. We have described how dynamic approaches became prominent at the dawn of this century, following the enticing perspective of finding self-fitted dynamic covalent receptors displaying high selectivity. The focus is now shifting towards the use of dissipative processes to produce fleeting receptors that exist transiently. Dissipative processes are often observed in biological systems^146^ and can be highly valuable for technological applications when it comes, for instance, to transport applications where recognition and release functions are combined within a single system able to bind guest only for a certain period before triggering release.^147^ In 2020, the Hartley group reported the first example of the transient formation of macrocycles (Fig. 10A).^148^ Their design involves the formation of anhydrides from the condensation of carboxylic acids 47 promoted by a dehydrating agent (EDC).^149^ Macrocycle 48 was found to transiently form over the course of a few hours with a magnitude and lifetime correlating with the amounts of starting material and chemical substrate being consumed (fuel). Aggregation was put forward to explain its accumulation prior to being slowly hydrolysed back to the starting material. In 2022, the Badjić group reported the transient formation of imine-based 3D molecular hosts using a self-degrading carboxylic acid that acts both as a Brønsted acid catalyst accelerating imine formation and as a templating guest (Fig. 10B).^150^ Covalent basket cage 52 was found to transiently form over the course of 2–12 hours in the presence of tribromoacetic acid fuel, which degrades by decarboxylation, causing a reorganization of the system through dynamic covalent exchange of the components 49–51.

Morphological changes^151^ can also be used to drive dynamic systems out of equilibrium. The Schmidt group used light as an external stimulus acting on an ortho-fluorinated azobenzene photo-switch to trigger the transient formation of imine macrocycles and temporal constitutional changes of the libraries.^152^ Earlier this year, the Feringa group has expanded this concept to light-induced cage interconversion, in which the photo-switching of an azobenzene unit promotes exchange between the different building blocks composing cage 53 (Fig. 10C).^153^ These approaches rely on the delicate relationship between the structural effect of photo-switching within closed molecular entities and the dynamic covalent exchanges that may follow. Imine exchange reactions can also be modulated by mechanical strain and torsion.^154^ Beyond the transient formation of discrete receptors, which is the topic of the present section, dissipative synthetic approaches are of interest to the design of smart materials and responsive supramolecular polymers.^155^ As final comment, the readers are warned that, as this field grows, the definition and terminology of these non-equilibrium steady state processes are currently being debated.^156^

## 10. Conclusions and perspectives

Dynamic covalent synthesis provides easy access to an astonishing variety of complex molecular architectures. It takes advantage of the reversible self-correction mechanisms occurring under thermodynamic control to promote the spontaneous amplification of target compounds. The simplicity of implementation makes this approach particularly elegant from a scientific point of view and appealing from economic and environmental perspectives: due to the associative nature of most reversible covalent reactions developed hitherto, DCS generally displays good atom economy. In addition, the combination of multiple reversible covalent reactions enables a remarkably precise integration of multiple components within a single product.^122^ The syntheses can be stereoselective,^34,57,83,157^ and post-synthetical conversion of the reversible covalent linkages into irreversible bonds can freeze the assemblies. The main limitation of DCS so far has been its relative lack of predictability. In recent years, however, an improved understanding of this type of systems has allowed successful programming of a growing number of building blocks.

Looking ahead, one can foresee major developments coming from molecular modelling approaches where simple tools can enable screening *in silico* the formation of cage-type structures which will be particularly informative for flexible systems.^158^ Combining such computational tools with robotic screening will significantly speed up the discovery process and expand the range of complex structures that can be produced.^159^ With the escalating impact of artificial intelligence,^160,161^ this growing body of data will improve the predictability of complex synthesis of discrete assemblies. Also, the templating approach now extends far beyond small spherical ions toward more complex and flexible biomolecular scaffolds^122,162^ and nano-templates.^163^ As for materials, DCS can be used as a bottom-up hierarchical approach to yield 2D and 3D (porous) materials^164–166^ such as Covalent Organic Frameworks (COF)^167–172^ and the alike Metal Organic Frameworks (MOF).^173,174^

Besides its great value as a modern synthetic methodology, dynamic covalent synthesis has a bright future as a tool towards increasingly sophisticated functional systems. Dynamic covalent assemblies respond to a wide range of physical and chemical triggers. Their responsive nature allows for bottom-up function manipulation and is of great interest to the development of smart recognition and delivery systems. Their responsiveness is also of great interest to the development of the next-generation dynamic and adaptative materials that will become part of a circular economy.^175^ Finally, dissipative processes provide a means to maintain dynamic covalent systems out of equilibrium. Out-of-equilibrium systems somehow mimic the behaviour of cell factories in nature by producing transient functional entities with defined lifetimes. The field is rapidly evolving in this direction, and new strategies are constantly emerging to enable endoergonic DCS to produce out-of-equilibrium products (*e.g.*, strategies based on energy ratchet mechanisms).^176,177^

The structural and functional complexity of the molecular assemblies potentially accessible by DCS is truly thrilling! The recent advances described in this review suggest it might become possible one day to design monodisperse multi-component macromolecular assemblies rivalling with biomolecules, in terms of precision in composition (sequence precision), three-dimensional structure, function… and beauty.

## Abbreviations

CD: Cyclodextrin
DCB: Dichlorobenzene
DBU: 1,8-Diazobicyclo(5.4.0)undec-7-ene
DCS: Dynamic covalent synthesis
DMF: Dimethylformamide
DMSO: Dimethyl sulfoxide
DTT: 1,4-Dithiothreitol
EDC: *N*-(3-Dimethylaminopropyl)-*N*′-ethylcarbodiimide hydrochloride
EDU: *N*-(3-Dimethylaminopropyl)-*N*′-ethylurea
HPLC: High-performance liquid chromatography
MS: Molecular sieves
PE: Phenyleneethynylene
SWCNT: Single-walled carbon nanotube
TBA: Tribromoacetic acid
TFA: Trifluoroacetic acid

## Author contributions

All authors contributed equally to conceptualization and writing.

## Conflicts of interest

There are no conflicts to declare.

## Supplementary Material
